# Cardiogenic shock among cancer patients

**DOI:** 10.3389/fcvm.2022.932400

**Published:** 2022-08-22

**Authors:** Anais Curtiaud, Clement Delmas, Justine Gantzer, Lara Zafrani, Martin Siegemund, Ferhat Meziani, Hamid Merdji

**Affiliations:** ^1^Université de Strasbourg (UNISTRA), Faculté de Médecine, Hôpitaux Universitaires de Strasbourg, Nouvel Hôpital Civil, Service de Médecine Intensive-Réanimation, Strasbourg, France; ^2^Intensive Cardiac Care Unit, Cardiology Department, University Hospital of Rangueil, Toulouse, France; ^3^Department of Medical Oncology, Strasbourg-Europe Cancer Institute (ICANS), Strasbourg, France; ^4^Medical Intensive Care Unit, Saint-Louis Hospital, Assistance Publique des Hôpitaux de Paris, University of Paris, Paris, France; ^5^Intensive Care Unit, Department of Acute Medicine, University Hospital, Basel, Switzerland; ^6^Department of Clinical Research, University of Basel, Basel, Switzerland; ^7^INSERM (French National Institute of Health and Medical Research), UMR 1260, Regenerative Nanomedicine (RNM), FMTS, Strasbourg, France

**Keywords:** heart failure, cardiogenic shock, cancer patient, cardio-oncology, cancer therapy

## Abstract

Sophisticated cancer treatments, cardiovascular risk factors, and aging trigger acute cardiovascular diseases in an increasing number of cancer patients. Among acute cardiovascular diseases, cancer treatment, as well as the cancer disease itself, may induce a cardiogenic shock. Although increasing, these cardiogenic shocks are still relatively limited, and their management is a matter of debate in cancer patients. Etiologies that cause cardiogenic shock are slightly different from those of non-cancer patients, and management has some specific features always requiring a multidisciplinary approach. Recent guidelines and extensive data from the scientific literature can provide useful guidance for the management of these critical patients. Even if no etiologic therapy is available, maximal intensive supportive measures can often be justified, as most of these cardiogenic shocks are potentially reversible. In this review, we address the major etiologies that can lead to cardiogenic shock in cancer patients and discuss issues related to its management.

## Introduction

**Figure 1:**
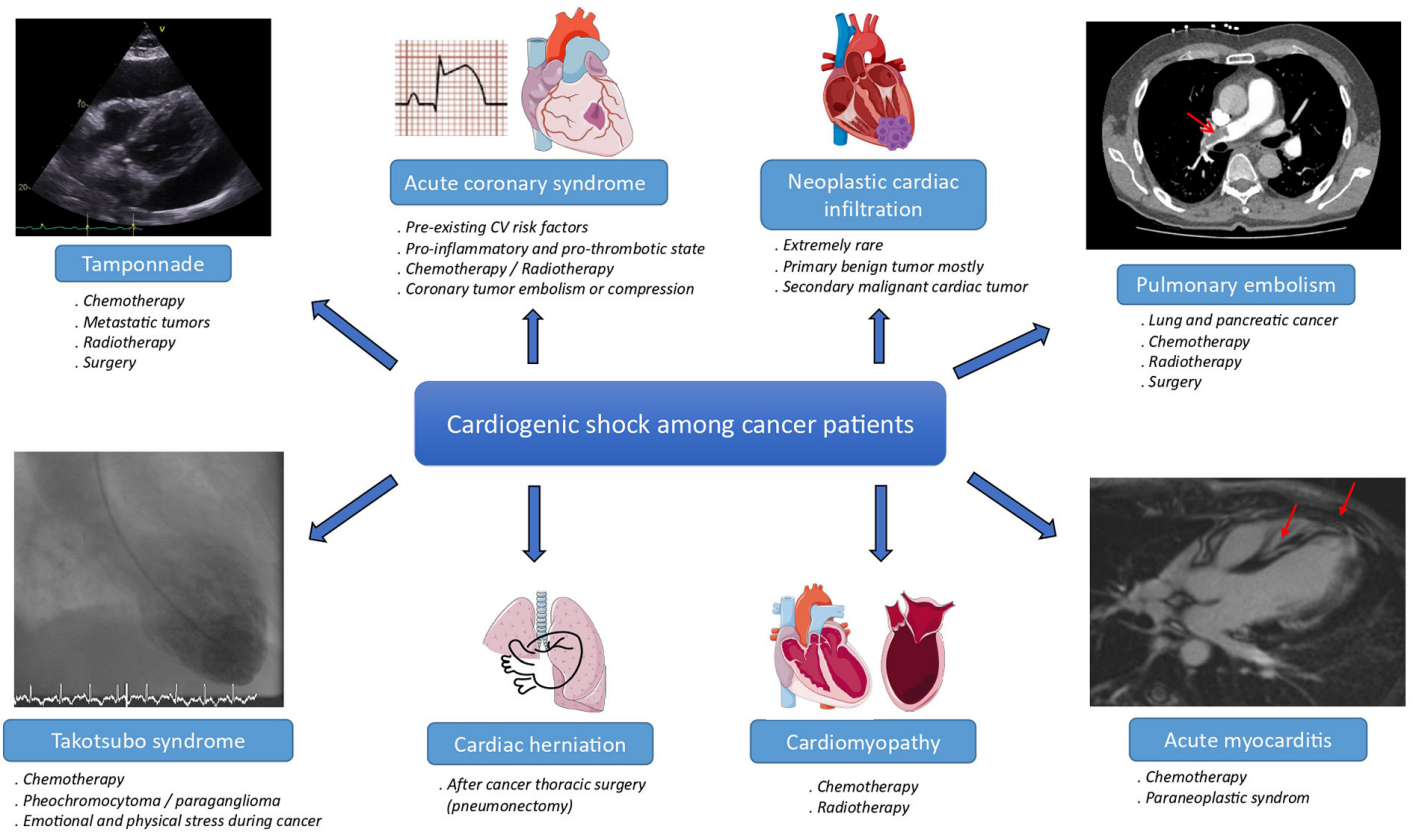
Main etiologies of cardiogenic shock among cancer patients. CV, cardiovascular. The meaning of the red arrow is “Magnetic resonance imaging of acute myocarditis”.

Cancer and cardiovascular diseases are the two most prevalent diseases worldwide and the leading cause of death in modern countries (1). However, due to advances in medicine, the mortality rate among cancer patients (CPs) has decreased dramatically over the last three decades (2, 3). Therefore, more of these CPs who also share many cardiovascular risk factors (4) develop fatal heart diseases (5), such as cardiogenic shock (CS) which can be caused by many distinct etiologies (Figure 1).

Cardiogenic shock may result from cancer itself, directly or indirectly through increased coronary risk and thromboembolic events, tamponade, or paraneoplastic syndrome. In these situations, CS belongs to cardio-oncology syndrome type one in the new classification of cardio-oncology syndromes published in the *Journal of the International Cardio-Oncology Society* (ICOS) (6). Cardiogenic shock may also result from collateral side effects of cancer treatments, which belong to cardio-oncology syndrome type two (6). The inflammation in cardio-oncology type two syndrome has been described as a hallmark of cancer therapy-induced cardiovascular complications, whether through an increase in proinflammatory cytokines such as interleukin-1 or inflammasome (7).

Cancer treatments are improving, but many therapies such as surgery, radiotherapy, chemotherapy, targeted therapies (e.g., hormone therapies, angiogenesis inhibitors), and immunotherapy might have significant cardiotoxicity. Consequently, cardio-oncology has emerged as a new and fast-growing subspecialty in recent years (8) with cardio-oncology teams and cardio-oncology services being progressively implemented in hospitals (9).

Cardiogenic shock is defined as a primary cardiac dysfunction with low cardiac output and without hypovolemia leading to critical organ hypoperfusion and tissue hypoxia. Diagnostic criteria include persistent hypotension and signs of compromised end-organ perfusion (10, 11). In recent times, CS remains one of the greatest challenges in cardiology and intensive care medicine. Compared to acute heart failure, CS has a 10-fold higher in-hospital mortality rate, remaining >40% despite medical and surgical advances (12). Given the specificities of different cancers or their treatments, management of CS in this particular setting requires a comprehensive knowledge of the various determinants in addition to a multidisciplinary collaboration between intensivists, cardiologists, cardiac surgeons, and oncologists. To help physicians evaluate and manage CP with acute cardiovascular disease, recent guidelines were published by a Task Force including the Association of Acute Cardiovascular Care (ACVC) and the Council of Cardio-Oncology (CO council) of the European Society of Cardiology (ESC) in 2021 (13).

In this review, we aim to summarize the latest evidence on CS among oncological patients to provide an overview of research areas and knowledge that are often at the crossroads of several medical specialties.

## Epidemiology

Epidemiology data underlying the relationship between CS and cancer are scarce, as most of the published data are case reports. In the largest European prospective multicenter study, which included CS patients from a broad spectrum of etiologies, 7% were CPs. In this registry, 30-day mortality was 29.4% in CPs. Interestingly, in this study, cancer was not an independent variable associated with 30-day mortality (14).

In contrast, in the administrative Nationwide Inpatient Sample Database which includes around 500,000 patients hospitalized for CS between 2004 and 2011, cancer was independently associated with poor outcomes. Having a solid tumor, with or without metastases, accounted for the most disadvantageous prognostic factors with an odds ratio (OR) of 2.05 and 1.50 respectively (15). In another study, based on the same database but for the period 2010 and 2014, CS was higher in patients with colon cancer and lower in patients with multiple myeloma, while patients with lung cancer had the highest risk of dying from CS (16).

### Cardiogenic shock related to acute coronary syndromes

In the largest published registry of acute coronary syndrome (ACS) in CP, the proportion of ACS among patients with cancer on treatment was ~3%. This number has been increasing since the early 2000s and the most common types of associated malignancies are lung, prostate, and breast cancers (17). The risk of ACS in patients with a current or historical diagnosis of cancer is more than two times higher as compared to the general population (18). Indeed, CPs are often older and may share traditional cardiovascular risk factors (4). In addition, cardiovascular toxicity induced by cancer therapies may cause ACS through different pathophysiological mechanisms (Table 1). Coronary vasospasm is one of the most described mechanisms and is typically caused by 5-fluoro-uracil (5-FU) or its prodrug capecitabine (19). Other mechanisms may include plaque rupture resulting from cisplatin and vinca alkaloids (20), or coronary thrombosis due to pro-inflammatory and prothrombotic conditions associated with increased platelet aggregability induced by specific cancer therapies (e.g., cisplatin, vascular endothelial growth factor (VEGF) signaling pathway inhibitors and cyclophosphamide) (21). Direct endothelial injury associated with accelerated coronary artery disease induced by radiotherapy (22) can cause ACS typically within 10 to 30 years following treatment but rarely during treatment (23). Moreover, mortality caused by ACS is higher in oncological patients (24, 25) with malignancy being considered an independent predictor of increased risk of repeated revascularization and stent thrombosis (17). Patients with ACS and malignancies might also be more likely to experience CS (26), even if this additional risk is not found in all studies (27).

**Table 1:** Main cancer therapies that can induce acute coronary syndrome in cancer patients.

**Therapy**	**Mechanisms**	**Time of onset**
**Antimetabolites** 5-FU Capecitabine	Vasospasm	Within 2 to 5 days
**Alkylating agents** Cisplatin	Oxidative stress, endothelial dysfunction	Within 3 months
**TKIs** Sunitinib Nilotinib	Endothelial, platelets and coagulation activation	Within 2 years
**Anti-microtubule agents** Paclitaxel Docetaxel	Vasospasm, cellular hypoxia	Within 2 weeks
**VGEF inhibitors**	Acute thrombosis	Within 3 months
**Radiotherapy**	Oxidative stress, fibrosis, and direct endothelial injury accelerated CAD	15–30 years following treatment

*TKIs, tyrosine kinase inhibitors; 5-FU, 5-fluorouracil; VEGF, vascular endothelial growth factor; CAD, coronary artery disease*.

Clinical presentation and diagnostic algorithms of ACS in CPs are relatively close to those in patients without cancer. However, ACS symptoms can frequently be atypical in CPs, mistaken with cancer symptoms such as cancer-related anemia (28), with less than one-third of them experiencing chest pain, and less than half of them having dyspnea (29).

Acute coronary syndrome management in CS remains almost identical to non-CPs, although sometimes complicated by increased comorbidities in CPs (30) or cytopenia. Chemotherapy-induced thrombocytopenia may be caused by DNA synthesis inhibition in megakaryocyte development, leading to megakaryocyte progenitor cell death due to alkylating agents or by oxaliplatin-dependent antibodies cross-reaction with platelet antigens (31). Consensus statements for ACS management in CPs advise early echocardiography to evaluate left ventricular function and exclude cancer or cancer therapy-related complications (13). Cardiotoxic cancer therapy should be at least temporarily interrupted after multidisciplinary discussion, especially if a causal relation is suspected (13). Experts advise that if a stent is needed, a drug-eluting stent (preferred over balloon angioplasty or bare metal stent) together with shortened dual antiplatelet therapy duration (mandatory for only 1 month for instance) is probably the safest choice in CPs undergoing percutaneous coronary intervention (PCI) who do not need short-term surgery. Aspirin and clopidogrel should be preferred to ticagrelor and prasugrel because of the high bleeding risk and limited data regarding both efficacy and safety in patients with active cancer (13). Thrombocytopenia due to cancer or cancer therapy is observed in about 10% of patients, which makes ACS management challenging because of an increased risk of bleeding complications. In CPs, experts advise that aspirin and clopidogrel can be administered if platelets are >10,000 and >30,000/μL, respectively. Experts also suggest that a minimum platelet count of 30,000 and 50,000/μL is required for PCI and coronary artery bypass grafting respectively (13).

### Cardiogenic/obstructive shock related to acute pulmonary embolism

Incidence of venous thromboembolic events (VTE) in CPs is around 15% (32). Cancer patients have an estimated seven-fold increased risk for VTE compared to the general population, especially in the first few months after cancer diagnosis (33). Thus, VTE is the second leading cause of death after cancer progression (34). Lung and pancreatic cancer are the most common malignancies associated with VTE (35, 36). In addition, lung and colorectal cancers are associated with the highest thromboembolic risk leading to pulmonary embolism (PE) (37).

The association between cancer and hypercoagulable state, sometimes eponymously referred to as Trousseau syndrome, has been known for more than a century. In addition to the usual risk factors, the hypercoagulable state of cancer is driven by activation of the coagulation cascade and platelet aggregation caused by tumor expression of procoagulant proteins released into the circulation such as tissue factor, plasminogen activator inhibitor-1, and podoplanin (38). Furthermore, cancer treatments such as surgery, hospitalization, central venous catheters, anti-tumor drugs [e.g., cisplatin, 5-fluorouracil (5-FU), tamoxifen], as well as supportive therapies (e.g., erythropoietin), may increase thrombosis via still incompletely understood mechanisms (39).

The risk of acute PE is therefore relatively high in CPs and its incidence is increased by surgery, chemotherapy, radiotherapy, and disease progression (40). In a retrospective study assessing the routine histologic examination of more than 1,300 surgical embolectomies in a general population, of whom 30% had a history of malignant disease, direct neoplastic emboli accounted for <1%. Among these <1% of patients, neoplastic embolism was the first manifestation of an underlying neoplasm in most cases, mainly caused by unknown lung cancer (41). This implies that CPs are much more likely to develop non-neoplastic thromboembolism than neoplastic emboli, although few rare cases of neoplastic arterial embolism leading to fulminant pulmonary hypertension complicated by right ventricular failure and cardiogenic shock have been identified (42, 43).

In a recent study, acute PE in CPs was associated with a 90% increase in all-cause inpatient mortality (44). These patients are less likely to present with chest pain (45) but most of them report dyspnea (40).

Guidelines recommend the use of thrombolysis for high-risk PE unless contraindicated (46). The only absolute contraindication for thrombolysis in cancer is central nervous system neoplasm, for which pulmonary embolectomy is recommended (46). Even though thrombolysis is not formally contraindicated in CPs, they are less likely to receive thrombolysis (47, 48) due to bleeding risk concerns and probably the intensity of cancer care.

Recent results on a retrospective database showed that surgical thrombectomy in patients with cancer is complicated with worse in-hospital outcomes, including mortality and more post-procedural bleeding than thrombolysis (49). Hence, another interesting option that should be considered, according to recent French and European guidelines, is embolectomy *via* percutaneous catheter-directed treatment for patients with high-risk PE in whom intravenous thrombolysis is contraindicated (46, 50).

Mortality due to PE is more important during the first 3 months but even CPs who survive beyond this period have an increased risk of death compared to the general population (51). However, with a median survival time of more than 2 years, maximal treatment intensity during the acute phase should be considered in CPs with PE (52), especially in young patients with few comorbidities and early-stage cancer.

Finally, cancer-associated arterial thromboembolism is a less frequent but key part of Trousseau syndrome. In the CATS cohort, a cohort of 1,880 patients with active newly diagnosed or relapsed cancer, the frequency of arterial thromboembolism was 2.6% during a median prospective observation time of 2 years (53). This was mainly caused by lung and kidney cancer. Thus, in CPs, the development of arterial thromboembolism has been reported to be associated with a three to five-fold increased risk of death (54).

### Cardiogenic shock related to acute cardiomyopathy

Cardiomyopathy-associated ventricular dysfunction is another typical clinical scenario that may lead to CS in CPs, mostly due to cardiotoxic anticancer agents. Numerous guidelines emphasize the need to identify patients with an increased risk of developing cardiovascular toxicity (55–57). Although slightly different, all definitions include patients with previous heart disease and abnormal left ventricular function, elevated cardiac biomarkers before initiation of anticancer therapy, prior mediastinal radiotherapy, and patients with prior or ongoing anthracycline treatment or HER2 (Human Epidermal Growth Factor Receptor-2) targeted agents such as trastuzumab and trastuzumab-derived antibody-drug conjugates (58).

Among chemotherapy drugs that are most likely to induce cardiotoxicity, anthracyclines (e.g., doxorubicin) have been used since the late 1950s to treat solid and hematologic cancers such as lymphoma, leukemia, sarcoma, and breast cancer. Anthracyclines are associated with several cardiovascular toxicities, including dilated cardiomyopathy with left ventricular systolic dysfunction leading to heart failure (59). In an Italian prospective study assessing more than 2,600 patients, the overall incidence of anthracycline-induced cardiotoxicity was 9% (60). This cardiotoxicity was dose-dependent, with the highest incidence observed during the first year after the completion of chemotherapy in 98% of the cases. However, early detection and heart failure therapy allow full or partial recovery in 82% of patients (60). Notably, in this study, the more severe the cardiotoxicity was, the more tendency there was for life-threatening arrhythmias or conduction disturbances requiring pacemaker implantation (60).

Anthracycline-associated cardiotoxicity is now thought to occur at the time of first exposure, a hypothesis supported by the finding of troponin release after administration, mainly as a consequence of increased intracellular Ca2+concentration, oxidative stress, DNA damage, and impairment of DNA repair through inhibition of the topoisomerase II, activation of cell senescence, and cell death (61). Recent evidence points to the involvement of many mechanisms mainly converging toward mitochondrial dysfunction (62).

In the case of anthracycline-induced CS, treatment is based on the empiric management of CS. Through antioxidant effects (*via* decreased NO production) and reduction of intracellular Ca2+ in cardiomyocytes, sodium-glucose cotransporter 2 (SGLT-2) inhibitors, also called gliflozins, may be a promising cardioprotective strategy in anthracycline-associated cardiotoxicity (63).

However, although described in case reports, rapid recovery of cardiac function appears to be rare (64); some case reports even reported bridges to recovery months after long-term cardiac support device implantation (65, 66).

Besides anthracyclines, anticancer agents including chemotherapies and certain targeted therapies have been associated with acute heart failure (Table 2). Indeed, the transmembrane receptor HER4 partners with HER2 in cardiomyocytes, the latter being a target in cancer therapy. Of note, a number of anti-HER2 agents are approved in HER2-positive breast [e.g., monoclonal antibodies, antibody-drug conjugates, and tyrosine kinase inhibitors (TKIs)] and gastric cancer (e.g., monoclonal antibodies, antibody-drug conjugates) (67). Among them, trastuzumab, a targeted therapy to the HER2 receptor in breast cancer and all its derivatives such as trastuzumab emtansine or trastuzumab deruxtecan, can be responsible for acute cardiac toxicity, which is usually reversible as opposed to the aforementioned anthracyclines (59). For patients receiving cardiotoxic chemotherapy, such as anthracycline or other anti-HER2 therapy, guidelines recommend a three-monthly left ventricular ejection fraction monitoring (68).

**Table 2:** Main cancer therapies that can induce cardiomyopathies in cancer patients.

**Therapy**	**Mechanisms**	**Time of onset**
**Anthracyclines** Doxorubicin	Oxidative stress-induced DNA damage activation of senescence and cell death	Within the first year
**Alkylating agents** Carboplatin Cisplatin Cyclophosphamide	Oxidative stress, endothelial dysfunction	Within 1 to 2 weeks
**Monoclonal Antibodies** Trastuzumab (anti-HER2)	Cardiomyocytes stunning and hibernation	Within 4 to 8 weeks
**TKIs** Imatinib Sunitinib Sorafenib	Oxidative stress, inhibition of NO cell apoptosis	Within the first year
**Proteasome inhibitors** Bortezomib	Not fully understood	Within 2 years
**Radiotherapy**	Oxidative stress, fibrosis and endothelial cell damage	15–30 years following treatment

*TKIs, tyrosine kinase inhibitors; HER2, human epidermal growth factor receptor-2; DNA, deoxyribonucleic acid; NO, nitric oxide*.

### Cardiogenic shock related to myocarditis

Myocarditis is an inflammatory condition leading to inflammatory cell infiltration into the myocardium. It can be a consequence of anticancer treatment (Table 3) or paraneoplastic syndrome, which can lead to CS in cancer patients (69).

**Table 3:** Main cancer therapies that can induce myocarditis in cancer patients.

**Therapy** **Time of onset**	**Mechanisms**	**Frequency/**
**Antimetabolites** 5-FU	Dysregulated inflammatory response	Extremely rare/At the beginning
**Alkylating agents** Cyclophosphamide	Not fully understood	Unknown/within 1-3 weeks
**ICIs** Ipilimumab (CTLA-4 inhibitor) Atezolizumab (PDL-1 inhibitor) Nivolumab (PD-1 inhibitor)	*T*-cells could target an antigen potentially shared by the tumor and cardiomyocytes	<1%/within the the first month

*ICIs, immune checkpoints inhibitors; 5-FU, 5-fluorouracil; CTLA-4, cytotoxic T-lymphocyte-associated protein 4; PDL-1, programmed death ligand-1; PD-1, programmed cell death protein-1*.

Identified many years ago, myocarditis secondary to the antimetabolite 5-FU is a very rare complication related to an inflammatory response, driven by apoptosis of myocardial and endothelial cells (70). Cyclophosphamide, a nitrogen mustard alkylating agent increasingly used to treat various types of cancers and autoimmune conditions, can rarely lead to myocarditis, occurring within 1–3 weeks, usually after high doses (>1.5 g/m^2^/day) (71).

Most recently, novel therapies harnessing the immune system, such as immune checkpoint inhibitors (ICI), have been proven to be associated with myocarditis. Although rare (less than 1%), it often occurs about 1 month after the first dose (72). ICI-related myocarditis usually appears in a fulminant presentation with a high fatality rate of almost 50% (73). The histopathological features of ICI-associated myocarditis imply myocardial infiltration of T-lymphocytes, both CD4+ and CD8+, and macrophages leading to myocyte death, without B-lymphocytes being present (74). A potential pathophysiological hypothesis for ICI-myocarditis is that cardiomyocytes may share targeted antigens with the malignancy, thus becoming targets of the same activated T-cells clones, leading to lymphocytic infiltration of the myocardium (75).

Besides cancer therapy, paraneoplastic syndromes are other possible triggers of myocarditis in CP. Catecholaminergic myocarditis has been associated with pheochromocytoma (76, 77), giant-cell myocarditis with lymphoma, sarcoma, lung cancer, and thymomas (78), and eosinophilic myocarditis has been associated in eosinophilic leukemia and lung cancer (79).

As in other causes of myocarditis, empirical treatments of myocarditis are often based on immunosuppressive therapies such as high-dose corticosteroids (61).

In specific ICI-associated myocarditis, high-dose intravenous corticosteroids and withdrawal of ICI are considered the first-line therapy (62), while abatacept (CTLA-4 agonist), alemtuzumab (anti-CD52 antibody), and anti-thymocyte globulin (anti-CD3 antibody) have been suggested in corticosteroid-resistant forms (63).

### Cardiogenic shock related to Takotsubo syndrome

Takotsubo syndrome (TTS) among CP has been mainly reported either as a cardiotoxic effect of antineoplastic treatment (Table 4), as a complication of specific tumors [such as pheochromocytoma and paraganglioma (80)], or as a complication of the significant emotional and physical stress that frequently accompanies cancer. TTS is a clinical syndrome that generally presents as chest pain mimicking ACS or as an acute heart failure marked by severe left ventricular systolic dysfunction mostly characterized by apical akinesis or ballooning with hyperdynamic basal segments, usually following an emotion or physical stressor, predominantly affecting post-menopausal women. TTS leading to CS occurs in ~10% of all cases according to the International Takotsubo Registry (InterTAK), which is the largest registry to date (81).

**Table 4:** Main cancer therapies that can induce Takotsubo syndrome in cancer patients.

**Therapy**
**Antimetabolites** 5-FU Capecitabine
**Monoclonal antibodies** Trastuzumab (anti-HER2) Rituximab (anti-CD20) Bevacizumab (anti-VEGF)
**TKIs** Sunitinib Ibrutinib
**ICIs** Ipilimumab (CTLA-4 inhibitor) Atezolizumab (PDL-1 inhibitor) Nivolumab (PD-1 inhibitor)
**Radiotherapy**

*TKI, tyrosine kinase inhibitors; ICI, immune checkpoint inhibitors; 5-FU, 5-fluorouracil; HER2, human epidermal growth factor receptor-2; VEGF, vascular endothelial growth factor; CTLA-4, cytotoxic T-lymphocyte-associated protein 4; PDL-1, programmed death ligand-1; PD-1, programmed cell death protein-1*.

Although the pathophysiology of the syndrome is still not well understood, commonly hypothesized mechanisms include circulating plasma catecholamines surge, inflammation, estrogen deficiency, microvascular dysfunction, and spasm of the epicardial coronary vessels (82). In the latest International Expert Consensus Document on Takotsubo Syndrome, malignancy, chemotherapy, and radiotherapy are listed among its triggers (83). 5-FU or its prodrug (capecitabine) have been involved in up to 50% of reported cases of TTS leading to CS (84). Its hypothetical mechanism may be coronary vasospasm and direct cardiotoxicity through the production of free radicals and microthrombi related to 5-FU–mediated kallikrein stimuli (84).

Interactions between TTS and malignancy are probably more complex than initially thought, as many studies now report that cancer will frequently be diagnosed within a few years following TTS. In a 4-year follow-up study, 9.6% of TTS patients developed malignancies (85) while Burgdorf observed cancer diagnosis in up to 14% of TTS patients in a 3-year follow-up study (86). These data suggest shared environmental or genetic triggers. Thus, reports have suggested variations in common signaling pathways related to survival cascades and cardioprotective roles which are thought to be up-regulated in adrenergic stress, such as the phosphatidyl inositol-3 kinase/protein kinase-B activation (87) and BCL2-associated athanogene3 protein polymorphism (88) as a potential link between cancer and TTS.

Prevalence of cancer in patients presenting with TTS has been reported to account for up to 28.5% (89). Therefore, cancer screening may be useful for patients who do not have a clear TTS-triggering stressor. In comparison to patients without cancer, the co-existence of cancer and TTS results in increased hospital length-of-stay with increased risk of mechanical ventilation (90, 91), cardiac arrest (92), and all-cause in-hospital and long-term mortality (93, 94). However, in the latest analysis of the InterTAK Registry, CPs with TTS did not experience more CS than non-CP (90).

Solid tumors appear more likely to develop TTS compared to hematologic malignancies (95). According to the InterTAK Registry, breast cancer was the most prevalent type of malignancy-related TTS in 26.2% of the cohort followed by tumors affecting the gastrointestinal system and the respiratory tract with a prevalence of 16.1% and 15.4%, respectively. Hematological malignancies were less prevalent than solid tumors, affecting up to 10% of patients (90). These differences are probably also partly due to the specificities of cancer treatment used for each cancer type.

In addition to the tumor type, the stage of cancer is also an important factor to be considered, as TTS appears to be more prevalent in patients with advanced or recurrent disease (95). Although, this could be explained by treatment selection bias.

Even if TTS in CPs remains poorly understood and multifactorial, several hypotheses are currently considered, such as the emotional trauma of the cancer diagnosis, the inflammatory state of cancer, and the physical stress of various cancer treatments (96, 97).

Guidelines regarding TTS management are lacking as no prospective randomized clinical trials have been performed in this patient population. Nevertheless, a recent international expert consensus endorsed by the ESC advises avoiding inotropes such as adrenaline, noradrenaline, dobutamine, and milrinone (83) because TTS patients treated with catecholamine drugs suffer a 20% increased mortality (98), although this may represent a selection bias due to the initial presentation of the patients. Instead, in TTS leading to CS, experts suggest considering the Ca2+-sensitizer levosimendan or short-term mechanical circulatory support (MCS) such as an axial flow pump (AFP) (e.g., impella) or venoarterial-extracorporeal membrane oxygenation (VA-ECMO) (83).

Experts also suggest looking for the presence of left ventricular outflow tract obstruction, which occurs in about 20% of TTS patients with CS (99). In this situation, experts suggest intravenous fluid, short-acting beta-blocker, and AFP to avoid diuretics, nitroglycerin, or intra-aortic balloon pump (IABP) (83).

Finally, pheochromocytomas and paragangliomas are rare neuroendocrine tumors that can cause catecholamine-induced myocardial dysfunction that may be complicated with CS in 2% of patients (76). To be differentiated from the classic phenotype of TTS, these conditions are labeled as TTS phenocopies (82) and may share genetic predispositions (100). Even though there are no guidelines, VA-ECMO support could be a life-saving therapy, allowing myocardial recovery within a few days (77). After hemodynamic stabilization, treatment should include α-blockade to negate the effects of the excess hormones secreted by the pheochromocytoma and minimize intraoperative hemodynamic instability, with elective tumor removal scheduled under stable conditions (77).

### Cardiogenic/obstructive shock related to cardiac tamponade

In 1935, thoracic surgeon Claude Beck first described the classic Beck triad in patients with acute cardiac tamponade including “hypotension, increased jugular venous pressure, and a small and quiet heart.”

In recent years, cancer represents ~25% of the cardiac tamponade etiology. Prognosis of cardiac tamponade is essentially related to the etiology, thereby patients with cancer and metastatic involvement of the pericardium usually have a bad short-term prognosis as it is the sign of advanced cancer. In a recent study, factors associated with poor prognosis at 2-years after pericardiocentesis for malignant effusions were age >65 years, platelet counts <20,000/μL, lung cancer, presence of malignant cells in the effusion, and drainage duration (101).

Pericardial effusion develops in up to 21% of patients with underlying malignancy (102). Cancers most frequently presenting with involvement of the pericardium are mainly solid malignancies, such as advanced lung cancer (~30% of patients with lung cancer present pericardial effusion), malignant melanoma (40–70% of patients), breast cancer (~25% of patients), and less frequently hematological malignancies such as leukemia and lymphomas (about 15% of patients) (103).

Most malignancy-related pericardial effusions are caused by direct or metastatic invasion of a non-cardiac tumor. Primary malignant pericardial mesotheliomas or cardiac synovial sarcomas are very rare (104). It has been suggested that the pathophysiology of non-neoplastic effusions in CPs is related to obstruction of the mediastinal lymphatic system by tumor infiltration, which can also result from radiotherapy-induced fibrosis, especially after chemoradiotherapy for lung and esophageal cancers (105).

Effusions can also be paraneoplastic as a result of pericarditis (106). Cancer treatments themselves may affect the pericardium indirectly by increasing the risk of opportunistic viral infections-causing pericarditis or directly by causing pericarditis as with radiation therapy (23), chemotherapy (e.g., cyclophosphamide, anthracyclines), targeted therapy (e.g., TKIs), or immunotherapy (107).

Consensus statements for cardiac tamponade management in CP recommend that immediate echo-guided pericardiocentesis should be preferentially performed (13). Indeed, in a recent clinical report, percutaneous pericardiocentesis with extended catheter drainage was safe and effective in CPs, including those with thrombocytopenia managed by platelet transfusion support (101). A prolonged drainage (2–5 days) together with intrapericardial instillation of sclerosing agents (e.g., bleomycin) is suggested by experts to reduce the risk of recurrences (13). Since surgical pericardiotomy is less effective in CPs and associated with more complications, experts suggest that it should be conducted only when a safe percutaneous approach is not possible (13). In case of recurrent effusions, experts advise the creation of a pericardial window, surgically or *via* percutaneous balloon pericardiotomy, to reduce the risk of repeated interventions by allowing drainage into an adjacent space, usually the pleura (13), even if the outcome is poor in these situations.

Experts advise treating acute malignant pericarditis in the same way as in non-CPs with non-steroidal anti-inflammatory drugs and colchicine in the absence of contraindications (13) to relieve symptoms and reduce the risk of relapse as well as to avoid the development of constrictive pericarditis (108). Even if pericardial effusion in ICI-related pericarditis is not often associated with hemodynamic compromise and tamponade (109), experts suggest additional treatment with methylprednisolone (1 mg/kg/day) while temporarily discontinuing the ICI (13).

Physicians should be aware that neoplastic tamponades appear to be at the greatest risk for effusive-constrictive pericarditis (110) and pericardial decompression syndrome compared to non-CPs (111). Pericardial decompression syndrome is a very rare but potentially fatal complication following pericardial drainage manifesting with paradoxical hemodynamic deterioration and/or pulmonary edema, commonly associated with ventricular dysfunction. The onset of this syndrome after the procedure varied widely, ranging from “immediate” to 48 h (111).

### Cardiogenic/obstructive shock related to cardiac herniation

Cardiac herniation is a very rare complication mainly encountered after cancer thoracic surgery with a high mortality rate (50–100%) (112). Though it was first reported in medical literature in 1948 after a pneumonectomy of the left lung for a carcinoma (113), this disease remains largely unknown (114).

Although it looks similar on both sides of the heart, the pathophysiology mechanism leading to hemodynamic failure is different (115). On the right side, the protrusion of the heart through an ignored or inadequately closed pericardial sac defect usually following a pericardiotomy may enable the heart to rotate its tip to the right around the superior vena cava/inferior vena cava axis, resulting in torsion of these large vessels and leading to a dramatic reduction of cardiac preload and thus cardiac output (116). On the left side, it involves protrusion and/or rotation of the left ventricular through the pericardial defect causing its strangulation (116).

Usually occurring within the first 24 h after surgery, a case report has described a sudden cardiac herniation up to 6 months after a right pneumonectomy (117). The only effective resuscitative treatment seems to be an emergency re-thoracotomy with the closure of the pericardial defect and restoration and fixation of the herniated heart to its normal position (118).

### Cardiogenic shock related to neoplastic cardiac infiltration

Primary cardiac tumors are extremely rare, with an autopsy frequency ranging from 0.001 to 0.03% (104). Most of these primary lesions are usually benign (119) but can also be malignant, such as cardiac sarcoma, which accounts for ~2% of primary cardiac tumors (120). In contrast, cardiac metastases are slightly more common (121), with up to 12% of CPs having metastases to the heart or pericardium at autopsy, although most of them remain clinically silent (122, 123). Thus, only 1% of total extracardiac malignancies have clinically symptomatic cardiac involvement, mainly caused by melanoma, lymphoma, leukemia, and carcinoma of the lung, breast, and esophagus (121, 124). The pathophysiology includes a direct extension (e.g., lung carcinoma), hematogenous seeding (e.g., melanoma, lymphoma), venous extension (e.g., renal carcinoma), and retrograde lymphatic seeding (e.g., breast carcinoma) (119).

Although the literature is full of case reports and autopsy studies, the prevalence of cardiac infiltration leading to CS is unknown. The reference treatment for primary cardiac tumors remains cardiac resection surgery, if possible, with few exceptions (125). Resection is usually not indicated for secondary malignant cardiac tumors and treatment mainly relies on other anticancer therapies (126).

## General issues for consideration regarding cardiogenic shock among cancer patients

Cancer and cardiovascular diseases are the leading causes of mortality worldwide. Evidence shows that these diseases have common risk factors, in an aging population, and are interconnected by adverse effects of cancer treatments on cardiovascular status (Table 5) (127). However, patients with cancer were excluded from most of the large cardiology studies and registries (128). Therefore, there is very little information on the impact of cancer in CS, even though data are emerging (5).

**Table 5:** Main etiologies related to cardiogenic shock in cancer patients.

	**Cancer disease**	**Chemotherapy**	**Targeted therapy**	**Immunotherapy**	**Radiotherapy**	**Surgery**
Acute coronary syndromes	X	X	X		X	
Acute pulmonary embolism	X	X			X	X
Acute cardiomyopathy		X	X		X	
Myocarditis	X	X		X		
Takotsubo syndrome	X	X	X	X		
Cardiac tamponade	X	X	X	X	X	X
Cardiac herniation						X
Neoplastic cardiac infiltration	X					

The occurrence of acute cardiovascular diseases in the cancer trajectory often causes interruption of potentially effective treatment, precluding completion of the therapy and influencing oncologic prognosis.

Core CS therapeutic principles do not differ substantially from non-CPs, even if there are some specificities such as those aforementioned. Complexity rather lies in the treatment intensity of CPs that can reasonably be implemented in the best interest of the patient.

**Figure 2:**
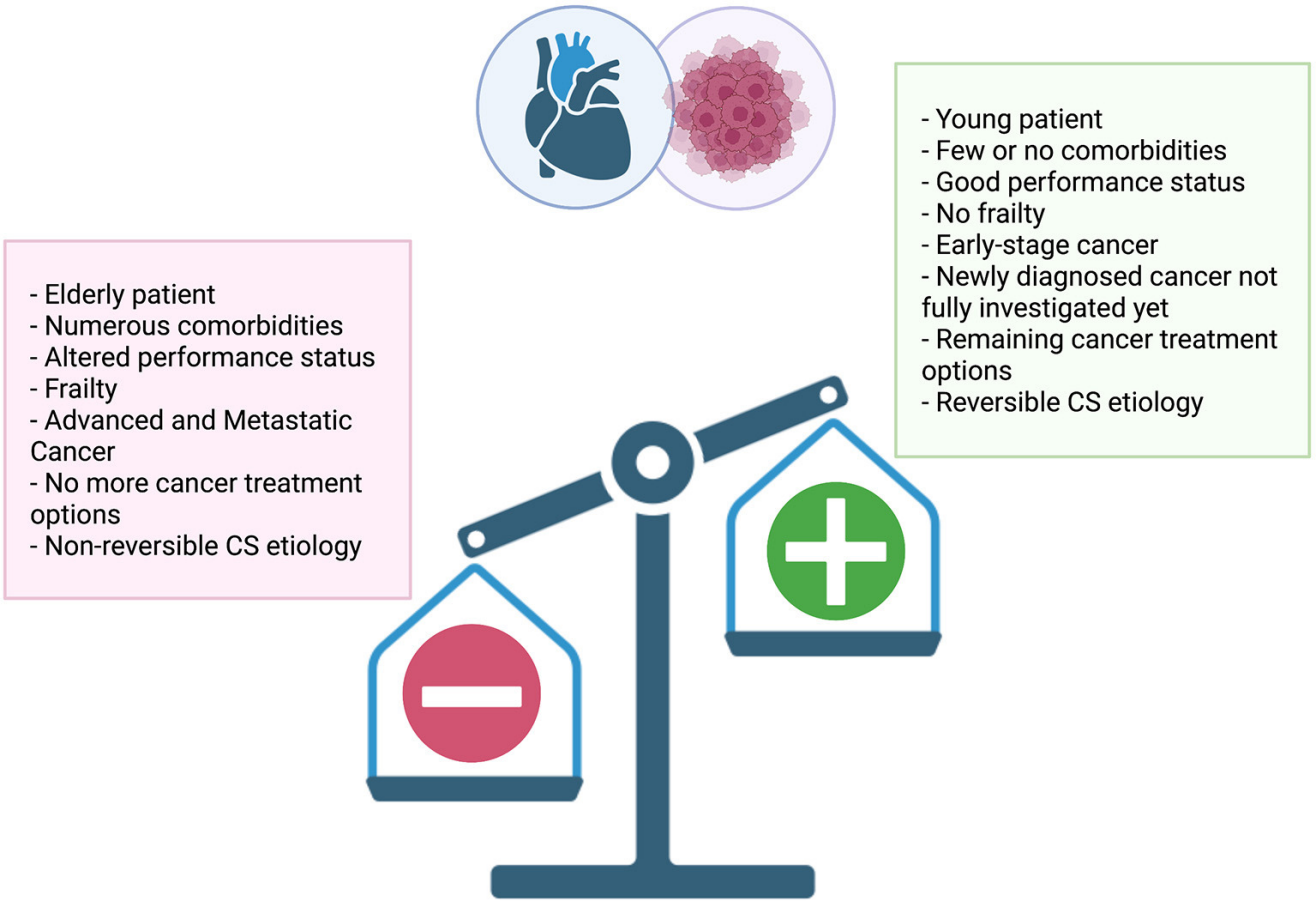
Multiple criteria to consider when assessing treatment intensity of cardiogenic shock in cancer patients.

According to a recent experts' review on critically ill oncology and hematology patients, no predefined criteria or prognostic scores of intensive care unit (ICU) or cardiac care unit triage for admission should be used (129). Each situation being different and challenging, the benefit-risk assessment must be discussed in an urgent multidisciplinary manner based on multiple criteria such as performance status (130) and frailty (131, 132) (Figure 2).

Experts also suggest that time-limited trials should be used for CPs, meaning unlimited ICU management with a full-code status for a limited period before a re-evaluation of the clinical situation (129).

The appropriate length of time for full-code status (doing everything that can be done, including cancer chemotherapy and short-term MCS) seems to be 1 week in CPs with solid tumors. To be noted, in the case of multiple organ failure, full-code management for 4 to 5 days leads to similar outcomes as unlimited aggressive care (133). Full-code status seems to be at least 2 weeks in hematology patients unless they are in multiple organ failure, in which case 1 week would be enough to provide the same survival as with unlimited aggressive care (133).

For instance, a full code status should be warranted in CS cases of newly diagnosed malignancies, PE in CPs with good performance status and no frailty, acute cardiac toxicity after complete cancer remission, and clinical response undetermined or still unpredictable (59). If needed, short-term MCS for refractory CS should be implemented before the onset of multi-organ failure in selected patients as a strategy to buy time for cardiac recovery (bridge-to-recovery strategy) or bridge-to-other therapies (bridge-to-decision strategy) (134).

Although evidence for the potential effect of cardioprotective drug therapy in preventing or mitigating the cardiotoxic effects of cancer therapy is incomplete to date, these therapies may play an important role in the future (135, 136).

## Conclusion

Cancer and cardiovascular diseases are the most prevalent diseases worldwide. Cardiogenic shock among cancer patients is an issue that can occur as a result of various causes and is likely to increase in the coming years. With improvements in cancer therapy and intensive care medicine, cardiogenic shock in cancer patients no longer means poor survival prognosis and initiation of palliative care. Rather, full code status with unlimited intensive care management (no restriction) is indeed worthwhile in some very speific situations. Multidisciplinary collaboration between intensivists, cardiologists, cardiac surgeons, and oncologists is essential in these critical situations.

## Author contributions

AC, FM, and HM wrote the manuscript, reviewed, and edited the article before submission. CD, JG, LZ, and MS revised the manuscript. AC and HM created the figure with BioRenders.com subscribed to HM. All authors approved the final manuscript.

## Conflict of interest

The authors declare that the research was conducted in the absence of any commercial or financial relationships that could be construed as a potential conflict of interest.

## Publisher's note

All claims expressed in this article are solely those of the authors and do not necessarily represent those of their affiliated organizations, or those of the publisher, the editors and the reviewers. Any product that may be evaluated in this article, or claim that may be made by its manufacturer, is not guaranteed or endorsed by the publisher.

## Abbreviations

5-FU: 5-fluoro-uracil
ACS: acute coronary syndrome
AFP: axial flow pump
CP: cancer patient
CS: cardiogenic shock
ESC: European Society of Cardiology
HER2: Human Epidermal Growth Factor Receptor-2
IABP: intra-aortic balloon pump
ICI: immune checkpoint inhibitors
ICOS: International Cardio-Oncology Society
InterTAK: International Takotsubo Registry
MCS: mechanical circulatory support
PE: pulmonary embolism
TKIs: tyrosine kinase inhibitors
TTS: Takotsubo syndrome
VA-ECMO: venoarterial-extracorporeal membrane oxygenation
VTE: venous thromboembolic events.
